# Recollections of General Grant

**Published:** 1890-09

**Authors:** 


### BOOK NOTICES.

Recollections of General Grant. By George W. Childs. Philadelphia : Collins Printing House. 1890.

This clever little 16mo volume of 104 pages is from the pen of no less a person than the foremost citizen of Philadelphia, and one of its most distinguished journalists.

There is no name more widely known; none held in more affectionate remembrance by his numerous beneficiaries than that of George W. Childs, editor and proprietor of *The Public Ledger*, of Philadelphia.

He had a long and intimate acquaintance with General Grant; was his counselor, friend, and admirer. Therefore, with his own splendid character and reputation for integrity, every word that he may utter or write concerning the dead General acquires at once a weighty historical value for its crystal-clear truth.

George W. Childs is himself a man whose lustrous name and deeds should be so enshrined, that their brightness may illuminate all posterity. This will be in great measure accomplished by the perpetuation of his incomparable newspaper. But there yet remains much to be done through the enthusiastic devotion to the attainment of this object of some one of his friends, who will strive to imitate the admirable result he has accomplished for his friend General Grant.

In the eloquent language of George William Curtis, when referring to Mr. Childs:

"The recollections of such a life are necessarily full of interest. They are especially pleasant, because they do not associate narrowness and hardness and meanness and selfish intrigue with success, but, on the contrary, the open hand and the open heart." W. M. J.

